# Parental knowledge and awareness of post-tonsillectomy complications and instructions: a cross-sectional study in Hail, Saudi Arabia

**DOI:** 10.3389/fped.2026.1806703

**Published:** 2026-07-30

**Authors:** Nada Mohammed Albeladi, Maha Atallah Alshammari, Wasayf Murdhi Alshammari, Mohammed Alateeq, Abdullah D. Alotaibi, Abdulaziz S. AlQahtani

**Affiliations:** 1 Faculty of Medicine, University of Hail, Hail, Saudi Arabia; 2 Department of Otolaryngology-Head and Neck Surgery, College of Medicine, University of Hail, Hail, Saudi Arabia

**Keywords:** health education, parents' awareness, pediatric surgery, postoperative care, postoperative complications, Saudi Arabia, tonsillectomy

## Abstract

**Introduction:**

Parental awareness of post-tonsillectomy complications and postoperative medication use is essential for safe home care and early recognition of complications, yet knowledge gaps remain common. This study evaluated parents’ knowledge of post-tonsillectomy complications and medications and identified factors associated with overall knowledge in Hail, Saudi Arabia.

**Methods:**

A cross-sectional study was conducted among parents in Hail, Saudi Arabia. Data were collected using a structured, self-administered questionnaire covering sociodemographic characteristics, knowledge items, sources of information, and experiences with preoperative counseling and postoperative instructions. An overall knowledge score was calculated by assigning one point for each correct response, with a cutoff of 60% used to classify knowledge as poor or good.

**Results:**

Overall parental knowledge was limited, with 743 parents (73.5%) classified as having poor knowledge and 268 parents (26.5%) demonstrating good knowledge. Knowledge gaps were most notable regarding postoperative bleeding, emergency management, and appropriate medication use. Higher knowledge levels were significantly associated with older age, higher education, prior experience with a child who had undergone tonsillectomy, receiving information from doctors, and adequate preoperative counseling and postoperative instructions. Gender was not significantly associated with knowledge.

**Conclusion:**

Parents demonstrated insufficient knowledge despite acknowledging the importance of medical education. Better preoperative counseling and postoperative guidance are needed to improve parental awareness and child safety after tonsillectomy.

## 1 Introduction

Tonsillectomy is one of the most frequently performed surgical procedures in pediatric practice worldwide, primarily indicated for recurrent tonsillitis and sleep-disordered breathing, including obstructive sleep apnea (1, 2). Despite advances in surgical techniques and perioperative care, tonsillectomy remains associated with a range of postoperative complications that may significantly affect children and their caregivers during the recovery period (1). Globally, tens of thousands of pediatric tonsillectomies are performed annually, with reports indicating that more than 50,000 procedures are conducted each year among children younger than 15 years of age in several healthcare systems (3). Although the procedure is generally considered safe and effective, postoperative morbidity continues to represent an important clinical and public health concern (4).

Pediatric tonsillectomy is commonly performed as a day-care surgery, with children discharged home after a short postoperative observation period. This practice aims to reduce healthcare costs, minimize hospital-acquired infections, and allow recovery in a familiar home environment (5). Consequently, the responsibility for postoperative monitoring and care is largely transferred to parents or primary caregivers (6). Caregivers are expected to recognize normal postoperative symptoms, identify warning signs of complications, manage medications correctly, and seek medical care promptly when necessary. However, this transition of responsibility can be challenging, particularly when parents lack adequate knowledge or confidence in managing postoperative care (7).

Post-tonsillectomy pain is one of the most common and distressing complications, often described as severe and prolonged in pediatric patients (8). Poor pain control may lead to reduced oral intake, dehydration, sleep disturbances, and delayed recovery. Other frequently reported complications include postoperative bleeding, nausea, vomiting, fever, halitosis, and, less commonly, infection (9–11). Post-tonsillectomy hemorrhage remains the most serious complication, accounting for a substantial proportion of emergency department visits and hospital readmissions following the procedure (12). Readmission rates after pediatric tonsillectomy have been reported to be approximately 6%, with bleeding, poor oral intake, pain, and vomiting being the leading causes (12, 13).

Several studies have highlighted deficiencies in parental knowledge regarding post-tonsillectomy care. A previous research study reported that a substantial proportion of parents are not adequately informed about how to manage postoperative complications, particularly bleeding, dehydration, and pain (14). In this survey, fewer than half of parents reported receiving clear instructions on managing post-tonsillectomy complications, despite recognizing the importance of such education. Inadequate parental knowledge has also been linked to preventable emergency department visits and unplanned hospital readmissions (13).

Understanding parental knowledge gaps and identifying factors associated with better awareness are essential for designing targeted educational strategies and improving postoperative outcomes. Therefore, this study aimed to assess the level of awareness and knowledge among parents in Hail, Saudi Arabia, regarding post-tonsillectomy complications and postoperative medication use.

## 2 Methodology

A cross-sectional study was conducted to assess parents' awareness and knowledge regarding post-tonsillectomy complications and postoperative instructions. The study was conducted in Hail, Saudi Arabia. Data were collected over a defined study period using an online self-administered questionnaire. The study population included fathers and mothers aged 18 years and older who were residents of Hail, Saudi Arabia. Parents of children in Hail who agreed to participate were eligible to participate. Parents younger than 18 years of age were excluded from the study. Participation was voluntary, and only parents who provided electronic informed consent were allowed to complete the questionnaire. The required sample size was estimated using the Raosoft® sample size calculator, assuming a 95% confidence level, a 5% margin of error, and an anticipated awareness level of 50%. Based on these assumptions, the minimum required sample size was approximately 1,000 participants. A convenience sampling technique was used to include eligible parents. The questionnaire link was distributed through social media platforms, including WhatsApp, X (formerly Twitter), Snapchat, and Instagram. Participants were encouraged to share the survey link to enhance recruitment and reach a wider segment of the target population.

Data were collected using a structured questionnaire developed after reviewing relevant literature on post-tonsillectomy complications and parental education. The questionnaire included several sections covering sociodemographic characteristics, the child’s history of tonsillectomy, the parents' awareness and knowledge of post-tonsillectomy complications, postoperative medication use, sources of information, and preoperative counseling and instructions. Knowledge-related items included questions on pain, bleeding, feeding difficulties, dehydration, fever, bad breath, immune effects, and appropriate postoperative medications. A formalized pain scale was not utilized to evaluate the participants’ children's discomfort. Rather, the assessment depended on the parents' subjective description of the pain level, categorized from mild to severe.

## 3 Data analysis

Data analysis was performed using the Statistical Package for the Social Sciences (SPSS), version 28 (IBM Corp., Armonk, NY, USA). Collected data were coded, entered, and cleaned before analysis to ensure accuracy and completeness. Categorical variables, including sociodemographic characteristics, sources of information, parental counseling, awareness items, and postoperative practices, were summarized using frequencies and proportions and presented in tables and figures for descriptive purposes.

Parents' awareness and knowledge regarding post-tonsillectomy complications and medications were assessed using a structured questionnaire. For knowledge assessment, each correct response was assigned a score of one, while incorrect and “I do not know” responses were assigned a score of zero. An overall knowledge score was calculated by summing the scores across all relevant items. The total score was then converted to a percentage, and a cutoff point of 60% of the maximum achievable score was used to classify the parents' knowledge level as poor (<60%) or good (≥60%).

A bivariate analysis was conducted to examine the association between parents' overall knowledge level and selected independent variables, including age, gender, educational level, child's history of tonsillectomy, sources of information, and preoperative counseling and instructions. The Pearson chi-square test was used to assess associations between categorical variables. When the assumptions of the chi-square test were not met, exact probability tests were applied as appropriate. Statistical significance was determined using a two-tailed *p*-value of less than 0.05.

## 4 Results

A total of 1,011 respondents completed the questionnaire. It is worth noting that the study population included parents whose children had not undergone tonsillectomy and those whose children had previously undergone the procedure. Table 1 shows the sociodemographic characteristics of the participating parents from Hail, Saudi Arabia (*N* = 1,011). The majority of the parents were aged between 36 and 45 years (387, 38.3%), followed by 334 participants (33.0%) aged 25–35 years. Furthermore, 170 individuals (16.8%) were younger parents aged less than 25 years, while parents older than 45 years were the smallest age group, with 120 participants (11.9%). As for gender distribution, women were more frequently represented than men, with 596 mothers (59.0%) compared to 415 fathers (41.0%). Regarding education level, nearly half of the respondents were university graduates (456, 45.1%), while 375 parents (37.1%) had completed middle or high school education. A smaller proportion of participants reported either no formal education or elementary education (75, 7.4%), and 105 parents (10.4%) held postgraduate degrees. Regarding clinical experience, 475 parents (47.0%) reported that their child had previously undergone tonsillectomy.

**Table 1:** Sociodemographic characteristics of the study participants (*N* = 1,011).

Sociodemographic characteristic	No.	%
Age in years		
<25	170	16.8
25–35	334	33.0
36–45	387	38.3
>45	120	11.9
Gender		
Male	415	41.0
Female	596	59.0
Education level		
None/elementary	75	7.4
Middle/high school	375	37.1
University graduate	456	45.1
Postgraduate degree	105	10.4
Has your child undergone tonsillectomy surgery?		
Yes	475	47.0
No	536	53.0

Table 2 presents parents' awareness and knowledge regarding post-tonsillectomy complications and postoperative medications among the study population in Hail, Saudi Arabia (*N* = 1,011). Overall, awareness varied across different domains, with generally higher recognition of pain-related complications compared to bleeding, fever, and knowledge related to medication. Regarding postoperative pain, 633 parents (62.6%) correctly identified pain as a common complication following tonsillectomy, while fewer than half recognized that postoperative pain can be severe (478 parents, 47.3%). More than half of the participants (578 parents, 57.2%) were aware that tonsillectomy may cause temporary pain and difficulty in swallowing. Awareness of reduced feeding and dehydration was lower, with 487 parents (48.2%) identifying these as common postoperative issues. Knowledge related to postoperative bleeding showed some gaps. Although 509 parents (50.3%) believed that bleeding is a common complication, responses were divided, and a substantial proportion reported uncertainty. When asked about personal experience, 338 parents (33.4%) reported that their child had experienced bleeding after surgery. Importantly, fewer than half of the respondents (457 parents, 45.2%) reported that they knew when to seek emergency care if postoperative bleeding occurred, and only 404 parents (40.0%) reported knowing how to manage bleeding at home, reflecting uncertainty in managing this potentially serious complication. With respect to other postoperative complications, 535 parents (52.9%) recognized that prolonged high fever after surgery may indicate complications, while awareness of bad breath as a possible postoperative issue was lower, reported by 463 parents (45.8%). Knowledge regarding the effect of tonsillectomy on the child's immunity was limited, as only 150 parents (14.8%) correctly indicated that tonsillectomy does not affect immunity, whereas a large proportion either believed it does or were uncertain. Concerning postoperative medication, the majority of the parents (704 parents, 69.6%) believed that both analgesics and antibiotics should be routinely administered after tonsillectomy. In contrast, only 147 parents (14.5%) correctly identified analgesia alone as the appropriate postoperative medication.

**Table 2:** Parents’ awareness and knowledge of post-tonsillectomy complications and medications in Hail, Saudi Arabia (*N* = 1,011).

Knowledge item	No.	%
Is pain a common complication after tonsillectomy?		
Yes	633	62.6
No	102	10.1
I don't know	276	27.3
Is pain after tonsillectomy severe?		
Yes	478	47.3
No	225	22.3
I don't know	308	30.5
Can tonsillectomy cause temporary pain and difficulty swallowing?		
Yes	578	57.2
No	118	11.7
I don't know	315	31.2
Is a decrease in feeding and dehydration common after tonsillectomy?		
Yes	487	48.2
No	157	15.5
I don't know	367	36.3
Is bleeding a common complication after tonsillectomy?		
Yes	509	50.3
No	163	16.1
I don't know	339	33.5
Did your child experience any bleeding after surgery?		
Yes	338	33.4%
No	360	35.6%
I don't know	313	31.0%
Do you know when to go to the emergency room if bleeding occurs after surgery?		
Yes	457	45.2
No	236	23.3
I don't know	318	31.5
Do you know how to manage bleeding at home?		
Yes	404	40.0
No	290	28.7
I don't know	317	31.4
Is a prolonged high fever after surgery a sign of complications?		
Yes	535	52.9
No	122	12.1
I don't know	354	35.0
Is bad breath a possible complication after tonsillectomy?		
Yes	463	45.8
No	136	13.5
I don't know	412	40.8
Does tonsillectomy affect the child's immunity?		
Yes	472	46.7
No	150	14.8
I don't know	389	38.5
What medication needs to be given after tonsillectomy?		
Analgesia	147	14.5
Antibiotics	78	7.7
Both of them	704	69.6
None	82	8.1

**Figure 1:**
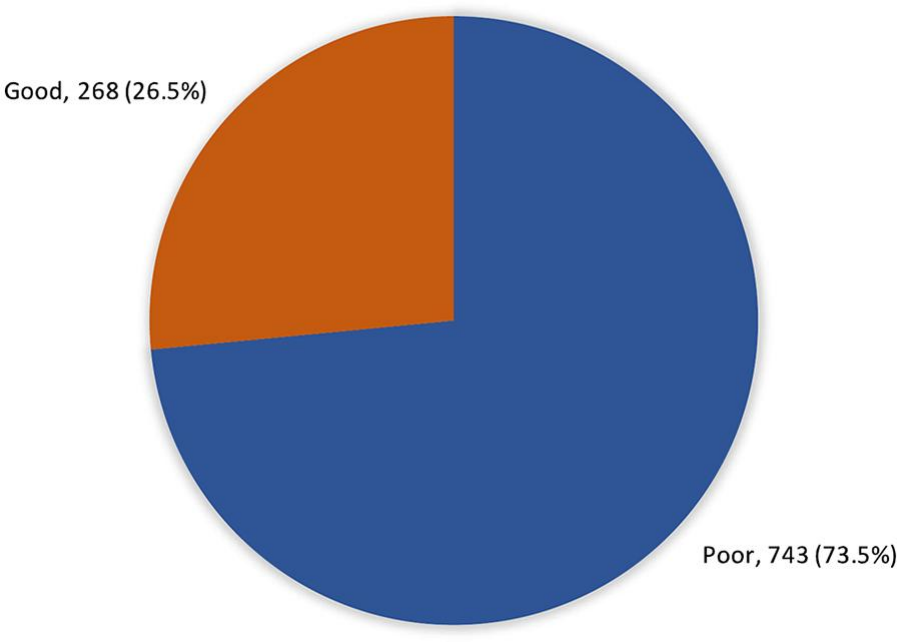
Parents’ overall awareness and knowledge of post-tonsillectomy complications and medications in Hail, Saudi Arabia (*N* = 1,011).

Figure 1 shows the overall level of parents' awareness and knowledge regarding post-tonsillectomy complications and postoperative medications among the study participants in Hail, Saudi Arabia (*N* = 1,011). The vast majority of the parents had a poor level of overall knowledge (743, 73.5%). In contrast, only 268 parents (26.5%) were classified as having a good level of awareness and knowledge.

**Figure 2:**
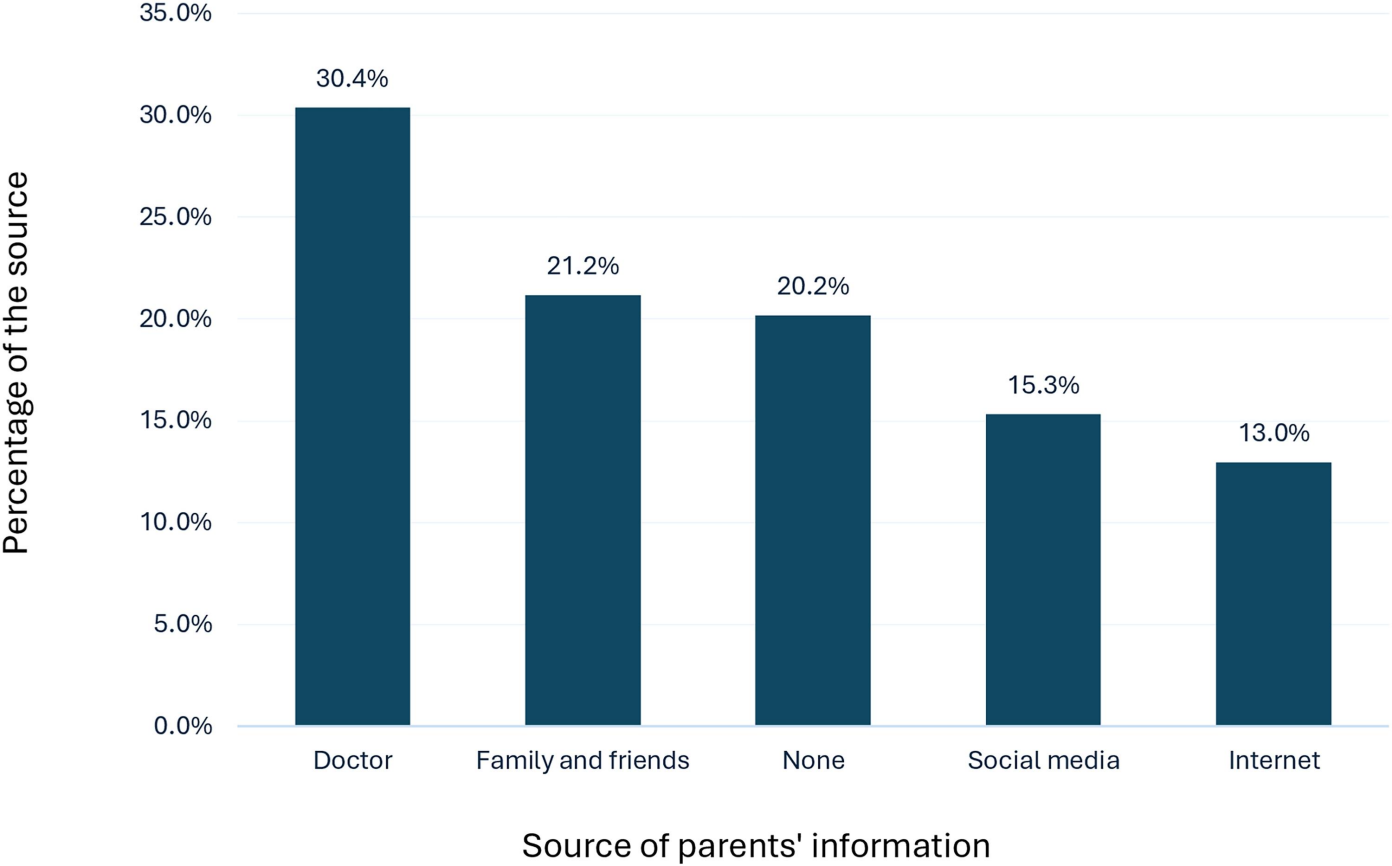
Sources of parents’ information about post-tonsillectomy complications in Hail, Saudi Arabia.

Figure 2 illustrates the main sources of information used by parents to learn about post-tonsillectomy complications in Hail, Saudi Arabia. Healthcare professionals were reported as the most common source, with 307 parents (30.4%) reporting doctors as their primary source of information. However, a considerable proportion of the parents relied on non-professional sources, including family and friends (214 parents, 21.2%), social media platforms (155 parents, 15.3%), and the internet (131 parents, 13.0%). Notably, 204 parents (20.2%) reported having no source of information at all regarding post-tonsillectomy complications.

Table 3 describes the parents' experiences with preoperative counseling, receipt of postoperative instructions, and their anticipated responses to post-tonsillectomy complications. Less than one-quarter of the parents reported that doctors explained possible complications in detail before surgery (236 parents, 23.3%), while 303 parents (30.0%) indicated that explanations were provided briefly. A notable proportion of the parents stated that no explanation was given (128 parents, 12.7%), whereas 344 parents (34.0%) reported that their child had not undergone tonsillectomy. Similarly, fewer than half of the parents (397, 39.3%) reported receiving instructions on how to manage postoperative complications. Despite these gaps, the majority of the parents strongly recognized the importance of preoperative medical education, with 767 parents (75.9%) agreeing that a doctor's explanation before surgery is essential for understanding complications. However, practical knowledge remained limited, as nearly half of the parents reported not knowing what to do if postoperative bleeding occurred (510 parents, 50.4%). In contrast, the majority of the parents demonstrated appropriate health-seeking behavior, as 880 parents (87.0%) stated they would consult a doctor if fever or pain persisted for several days after surgery.

**Table 3:** Parental counseling, preoperative education, and response to post-tonsillectomy complications among parents in Hail, Saudi Arabia (*N* = 1,011).

Knowledge item	No.	%
Did the doctor explain the possible complications before the surgery?		
Yes, in detail	236	23.3
Yes, briefly	303	30.0
No	128	12.7
My child did not undergo the surgery	344	34.0
Did you receive instructions on how to manage complications?		
Yes	397	39.3
No	249	24.6
My child did not undergo the surgery	365	36.1
Do you think a doctor's educational briefing before surgery is important to understand complications?		
Yes	767	75.9
No	54	5.3
I don't know	190	18.8
If bleeding occurs after surgery, do you know what to do?		
Yes	501	49.6
No	510	50.4
If fever or pain continues for several days, will you consult the doctor?		
Yes	880	87.0
No	131	13.0

Table 4 outlines parents' knowledge and reported practices regarding the use of analgesics following pediatric tonsillectomy in Hail, Saudi Arabia. Among parents whose children underwent the procedure, combined use of paracetamol and ibuprofen was the most frequently reported analgesic regimen, accounting for 302 parents (29.9%), while smaller proportions reported using paracetamol alone (94 parents, 9.3%) or ibuprofen alone (79 parents, 7.8%). Slightly more than half of the respondents (536 parents, 53.0%) indicated that their child had not undergone tonsillectomy. Regarding the duration of analgesic use, responses varied, with 185 parents (18.3%) reporting use for 7–10 days, followed by 134 (13.3%) reporting 5–7 days and 96 (9.5%) reporting 3–5 days. A smaller proportion reported extending analgesic use beyond 10 days (46 parents, 4.5%). Considering dosing frequency, paracetamol was most commonly reported as being given on an as-needed basis (236 parents, 23.3%), while fewer parents adhered to fixed dosing schedules, such as three times daily (78 parents, 7.7%) or four times daily (53 parents, 5.2%).

**Table 4:** Parental awareness and practices regarding post-tonsillectomy analgesic use among children in Hail, Saudi Arabia (*N* = 1,011).

Knowledge item	No.	%
What analgesia is given to your child?		
Paracetamol	94	9.3
Ibuprofen	79	7.8
Both of them	302	29.9
My child did not undergo the surgery	536	53.0
For how long should the medication be used?		
3–5 days	96	9.5
5–7 days	134	13.3
7–10 days	185	18.3
>10 days	46	4.5
My child did not undergo the surgery	550	54.4
How many times is paracetamol given per day?		
Once daily	56	5.5
Twice daily	52	5.1
Three times daily	78	7.7
Four times daily	53	5.2
As needed	236	23.3
My child did not undergo the surgery	536	53.0

Table 5 reveals the factors associated with parents' overall knowledge and awareness regarding post-tonsillectomy complications and medications. A statistically significant association was observed between age and overall knowledge level (*p* = 0.017), with a higher level of knowledge reported among parents aged 36–45 years (31.5%) compared with those aged <25 years (20.0%) and 25–35 years (23.7%). Gender was not significantly associated with knowledge level (*p* = 0.563), as comparable proportions of good knowledge were observed among men (27.5%) and women (25.8%). Education level showed a significant relationship with knowledge (*p* = 0.029). Parents who were university graduates demonstrated a higher proportion of good knowledge (30.7%) compared with those with middle or high school education (21.6%). Parents with no or elementary education (28.0%) and those with postgraduate degrees (24.8%) showed intermediate levels of knowledge. A strong association was also found between having a child who underwent tonsillectomy and parental knowledge (*p* = 0.001), as parents with previous experience reported considerably higher knowledge levels (41.7%) compared with those whose children had not undergone the procedure (13.1%). Sources of information were significantly associated with knowledge level (*p* = 0.001). Parents who cited doctors as their main source of information demonstrated the highest proportion of good knowledge (42.3%), whereas parents who reported having no source of information showed the lowest proportion of good knowledge (5.4%). Reliance on social media (31.0%) and the internet (27.5%) was associated with moderate knowledge levels, while information obtained from family and friends was linked to less knowledge (20.1%). Furthermore, a detailed preoperative explanation by doctors was strongly associated with better knowledge (*p* = 0.001), with nearly half of parents who received detailed explanations showing good knowledge (47.5%), compared with only 10.9% among those who reported receiving no explanation. Similarly, receiving instructions on how to manage postoperative complications was significantly associated with improved knowledge (*p* = 0.001). Parents who received instructions had a markedly higher proportion of good knowledge (46.3%) compared with those who did not receive instructions (11.6%).

**Table 5:** Factors associated with patients’ knowledge and awareness about post-tonsillectomy complications and medications.

Factor	Overall knowledge level				*p*-Value
	Poor		Good		
	No	%	No	%	
Age in years					0.017*
<25	136	80.0	34	20.0	
25–35	255	76.3	79	23.7	
36–45	265	68.5	122	31.5	
>45	87	72.5	33	27.5	
Gender					0.563
Male	301	72.5	114	27.5	
Female	442	74.2	154	25.8	
Education level					0.029*
None/elementary	54	72.0	21	28.0	
Middle/high school	294	78.4	81	21.6	
University graduate	316	69.3	140	30.7	
Postgraduate degree	79	75.2	26	24.8	
Has your child undergone tonsillectomy surgery?					0.001*
Yes	277	58.3	198	41.7	
No	466	86.9	70	13.1	
Sources of information and education					0.001*
Doctor	177	57.7	130	42.3	
Internet	95	72.5	36	27.5	
Family and friends	171	79.9	43	20.1	
Social media	107	69.0	48	31.0	
None	193	94.6	11	5.4	
Did the doctor explain the possible complications before the surgery?					0.001*^
Yes, in detail	124	52.5	112	47.5	
Yes, briefly	214	70.6	89	29.4	
No	114	89.1	14	10.9	
My child did not undergo the surgery	291	84.6	53	15.4	
Did you receive instructions on how to manage complications?					0.001*
Yes	213	53.7	184	46.3	
No	220	88.4	29	11.6	
My child did not undergo the surgery	310	84.9	55	15.1	

P: Pearson *X*^2^ test.

^ Exact probability test.

*P < 0.05 (significant).

## 5 Discussion

This study aimed to comprehensively assess parents' awareness and knowledge regarding post-tonsillectomy complications and postoperative care in Hail, Saudi Arabia. Overall, the findings showed extensive gaps in parental knowledge, despite the high education level of a considerable proportion of participants.

The parents showed moderate awareness of pain after tonsillectomy. Approximately two-thirds knew that pain is a common post-tonsillectomy complication, yet fewer than half knew that pain can be severe. Similar findings have been reported internationally, where parents often underestimate the severity and duration of post-tonsillectomy pain in children (7, 15). Levy et al. (15) showed that parental underestimation of pain severity is associated with suboptimal pain management and increased parental anxiety. This knowledge gap may contribute to inadequate analgesic use and delayed recovery, reinforcing the need for explicit counseling on expected pain trajectories.

Awareness of swallowing difficulty, reduced feeding, and dehydration was also unsatisfactory in the current study. Less than half of the parents identified decreased feeding and dehydration as common postoperative issues. These findings concur with previous studies that revealed poor oral intake is one of the leading causes of emergency department visits and hospital readmissions following tonsillectomy (3, 12). Tran et al. (16) reported that dehydration and pain are among the most preventable causes of post-tonsillectomy hospital revisits, often linked to insufficient caregiver education. Improving parental recognition of these complications could therefore play a key role in reducing avoidable healthcare utilization.

The parents’ lack of knowledge related to postoperative bleeding was concerning. Although bleeding is a well-recognized complication, only approximately half of the parents identified it correctly, and fewer than half reported knowing when to seek emergency care. These findings are consistent with a local Saudi study conducted by Alkhars et al. (14), which showed that many parents lack adequate knowledge about post-tonsillectomy hemorrhage and its management, despite considering it the most feared complication. Internationally, Jain et al. (12) found that inadequate parental education about bleeding was associated with higher rates of emergency department returns. Given that post-tonsillectomy hemorrhage can be life-threatening, these gaps represent a critical area for intervention.

Awareness of prolonged fever as a warning sign of complications was moderate, while knowledge of bad breath as a common postoperative finding was relatively low. Halitosis is generally a benign and temporary consequence of tonsillar wound healing (17), yet parental unfamiliarity with this symptom may lead to unnecessary anxiety and medical consultations. Similar misconceptions have been documented in qualitative studies exploring parents' postoperative experiences, where lack of anticipatory guidance contributed to stress and uncertainty (7, 18, 19).

An interesting finding was the widespread misconception regarding the effect of tonsillectomy on immunity. Only a small proportion of parents correctly recognized that tonsillectomy does not significantly impair long-term immune function. This misconception has been reported in both regional and international societies, reflecting persistent concerns among caregivers about the immunological role of the tonsils (20, 21). Addressing this belief during preoperative counseling may help reduce parental hesitation and postoperative anxiety.

Knowledge related to postoperative medications was among the weakest domains identified. The majority of parents believed that antibiotics should be routinely administered in addition to analgesics. This finding is consistent with previous studies reporting inappropriate expectations regarding antibiotic use after tonsillectomy (22, 23). Evidence-based guidelines do not recommend routine antibiotic use, as it does not significantly reduce pain, bleeding, or infection rates and may increase adverse effects (24, 25). Misunderstanding in this area underscores the need for clear, guideline-based medication counseling.

The overall knowledge assessment revealed that nearly three-quarters of the parents had poor knowledge, a finding that reflects results from other regional and international studies (7, 14). This widespread deficiency highlights that current educational practices are insufficient to ensure parental preparedness for home-based postoperative care.

Doctors were identified as the primary source of information; however, a considerable proportion of the parents trusted informal sources such as family, friends, social media, and the internet, or reported having no information source at all. Reliance on non-professional sources has been associated with inconsistent and sometimes inaccurate knowledge in previous studies (14, 20). Importantly, parents who cited doctors as their main source demonstrated significantly better knowledge, reinforcing the central role of healthcare professionals in effective patient education.

Preoperative counseling and postoperative instructions were strong determinants of parental knowledge. Parents who received detailed explanations and clear instructions were significantly more knowledgeable than those who did not. These findings are consistent with international evidence demonstrating that structured preoperative education reduces parental anxiety, improves pain management, and lowers rates of emergency department returns (12, 20). Duvenage et al. (5) further emphasized that caregiver education programs tailored to postoperative challenges can significantly enhance confidence and care quality.

Finally, prior experience with tonsillectomy was strongly associated with better parental knowledge, suggesting experiential learning plays an important role. However, reliance on experience alone is insufficient and may preserve misconceptions if not guided by accurate medical information. Age and education level were also associated with knowledge, while gender was not.

## 6 Study limitations

This study has some limitations that should be considered when interpreting the findings. First, the cross-sectional design limits the ability to infer causal relationships between parents' knowledge and the identified associated factors. Second, data were collected using a self-administered questionnaire, which may be subject to recall bias, principally for questions related to previous counseling and postoperative practices. Third, the study was conducted in a single region (Hail, Saudi Arabia), which may restrict the generalizability of the results to other regions with different healthcare systems or sociodemographic characteristics. In addition, although the questionnaire covered key aspects of post-tonsillectomy complications and medications, it did not explore parents' actual behaviors during real postoperative events, nor did it assess healthcare providers' perspectives or the quality of counseling delivered.

## 7 Conclusions and recommendations

In summary, this study demonstrates that parental awareness of post-tonsillectomy complications and postoperative care in Hail, Saudi Arabia, remains inadequate, particularly regarding bleeding management, medication use, and immune-related misconceptions. The findings emphasize the urgent need for standardized, comprehensive, and physician-led educational interventions delivered before and reinforced after surgery. Addressing these gaps has the potential to improve postoperative outcomes, reduce avoidable complications, and enhance parental confidence in caring for children following tonsillectomy.

## Data Availability

The datasets presented in this study can be found in online repositories. The names of the repository/repositories and accession number(s) can be found below: https://docs.google.com/spreadsheets/d/1MY81aHViuC5D2HQhwUVmYJbG6w34pYdR/edit?usp=sharing&ouid=110346998477386144132&rtpof=true&sd=true.
